# *Limosilactobacillus fermentum* MG7011: An Amylase and Phytase Producing Starter for the Preparation of Rice-Based Probiotic Beverages

**DOI:** 10.3389/fmicb.2021.745952

**Published:** 2021-09-29

**Authors:** Yu Mi Jo, Ga Yun Kim, Seul-Ah Kim, Seong Won Cheon, Chang-Ho Kang, Nam Soo Han

**Affiliations:** ^1^Brain Korea 21 Center for Bio-Health Industry, Department of Food Science and Biotechnology, Chungbuk National University, Cheongju, South Korea; ^2^MEDIOGEN, Co., Ltd., Seoul, South Korea

**Keywords:** rice, probiotics, *Limosilactobacillus fermentum*, amylase, phytase

## Abstract

The goal of this study was to develop a starter strain of *Limosilactobacillus fermentum* which is beneficial for human health and suitable for rice fermentation. To achieve the goal, the characteristics of 25 strains of *L. fermentum* were compared in terms of health promoting potentials and rice fermenting abilities. *L. fermentum* MG7011 was selected as a superior strain to meet the required properties. First, as probiotic traits, the strain had tolerance to gastrointestinal conditions and ability to adhere to Caco-2 and HT-29 cells. The strain showed the antioxidative activity, anti-inflammatory activity, and a protective effect on the epithelial barrier. Next, as starter traits for rice fermentation, MG7011 exhibited proper fermentation profiles in rice solution, such as fast growth rate, pH and metabolite changes, amylase and phytase activities, and optimal viscosity changes for beverage. In conclusion, *L. fermentum* MG7011 has excellent probiotic activities and proper starter traits in rice, thereby it can be used as a suitable probiotic starter for rice fermentation.

## Introduction

Cereals are consumed as common staple foods around the world – steamed rice in East Asia, wheat or barley breads in Europe and North America, and sorghum and maize-based gruel in Africa and South America (Tamang et al., 2020). These cereals are rich in carbohydrates, fibers, proteins, vitamins, and minerals and have been used as the major substrate of fermentation for a long time (Tangyu et al., 2019). Fermented cereals have improved nutritional value, sensory qualities, digestibility, and shelf life compared to unfermented cereals (Ghosh et al., 2015). In addition, cereals act as nutrient sources for the growth of beneficial bacteria, lactobacilli and bifidobacterial. Therefore, they are considered as the best alternative as non-dairy probiotic foods (Salmerón, 2017). The first non-dairy probiotic product was the fermented oatmeal beverage ‘ProViva’ with *Lactiplantibacillus plantarum* 299v, released in 1994 by a Swedish company (Maria et al., 2020).

Rice (*Oryza sativa L.*) is one of the best candidates for non-dairy probiotic providing rich nutrients and fibers (Sen et al., 2020). Fermented rice has metabolites such as phenolics, flavonoids, anthocyanins, phytosterols, linolenic acid, and γ-aminobutyric acid (GABA), which have various health-promoting effects, such as antioxidant and anticancer activities (Phutthaphadoong et al., 2009; Ray et al., 2016; Giri et al., 2018), and relaxing effects to help sleep disturbance (Mabunga et al., 2015). However, phytic acid, a six-fold dihydrogen phosphate ester of inositol, is reported a s an antinutrient because it forms complexes with minerals and proteins, inhibiting the uptake of nutrients in the intestines (Reale et al., 2004). Fermentation is an effective method for improving nutritional value because phytic acid can be degraded by bacterial phosphatases such as phytase (Naghmouchi et al., 2020). Previous studies have reported that certain lactic acid bacteria have phytase enzymes that catalyze the degradation of phytic acid in cereals during fermentation; wheat dough fermented by *Lactiplantibacillus plantarum*, *Levilactobacillus brevis*, *Latilactobacillus curvatus*, and *Limosilactobacillus fermentum* significantly decreased the phytic acid concentration (Reale et al., 2004).

Lactic acid bacteria play important roles in cereal fermentation not only for health benefits by producing organic acids, oligosaccharides, and polyphenolic compounds, but also for better flavor by producing volatile compounds (Ghosh et al., 2015). In addition, the cereals fermented with lactic acid bacteria often supplement limited level of amino acids such as methionine and lysine which are essential amino acids for human (Oguntoyinbo and Narbad, 2015). Among various lactic acid bacteria, *Limosilactobacillus fermentum* is regarded as one of the most adapted to cereal environment, because this species can utilize the abundant nutrients by enzymes such as amylase, feruloyl esterase, and phytase (Deng et al., 2019; Sharma et al., 2020). In addition, *L. fermentum* was reported as the most pre-dominant bacteria in Chinese cereal gruel (Qin et al., 2016), West Africa cereal dough (Houngbédji et al., 2018), and Indian rice-based fermented beverage (Ghosh et al., 2015). Furthermore, they are regarded as “generally recognized as safe” (GRAS) by the United States Food and Drug Administration (FDA) (FDA, 2013). With above research background, several studies have been conducted to isolate *L. fermentum* as a rice starter: amylolytic *L. fermentum* strains from African maize sourdough (Agati et al., 1998) and the KKL1 strain exhibiting α-amylase and glucoamylase activities (Ghosh et al., 2015). However, no study has been performed to isolate a strain exhibiting dual traits as a starter for rice fermentation and as a probiotic for human health.

In this study, we aimed to select a strain of *L. fermentum* which is beneficial for human health and suitable for rice fermentation. To this end, we isolated 25 different strains of *L. fermentum* from various plant-based fermented foods and compared their gastrointestinal stability (acid and bile tolerance, adhesion to intestinal epithelial cells), safety (biogenic amine-producing genes, hemolytic and phytase activities), and health-promoting activities (anti-inflammatory and antioxidant activities, and enhancement of epithelial barrier function). Then, against the three selected isolates, we analyzed their biochemical characteristics, fermentative profiles, and metabolites produced in rice solution. As a result, we selected *L. fermentum* MG7011 as the best strain for the role of a probiotic to provide anti-inflammatory activity as well as a starter to confer phytase activity and proper rheological properties in fermented rice.

## Materials and Methods

### Microorganisms and Culture Conditions

Total 25 strains of *Limosilactobacillus fermentum* (≥99.85% identity) were isolated from various plant-based fermented foods and they were used for this study. The type strain, *L. fermentum* DSM 20052 (LFT), was obtained from KACC (Korean Agriculture Culture Collection, Wanju, South Korea) and a commercial strain, *L. fermentum* KCCM 35469 (LFC), was from KCCM (Korean Culture Center of Microorganisms, Seoul, South Korea). *Lactiplantibacillus plantarum* WCFS1 and *Lacticaseibacillus rhamnosus* GG (LGG) were used as reference probiotics. All lactic acid bacteria were cultured in MRS broth at 37°C for 24 h.

### Probiotic Activity Tests

#### Acid and Bile Salt Tolerance Assay

Resistance to acidic conditions was tested according to the method of Conway et al. (1987). Lactic acid bacteria were cultured in MRS medium overnight and harvested by centrifugation at 6,000 × *g* for 10 min. Cells were washed twice with phosphate-buffered saline (PBS; pH 7.2) and resuspended in an equal volume of PBS adjusted to pH 3.0 and 2.5 with HCl. Following incubation for 0, 90, and 180 min at 37°C, acid tolerance was evaluated by spreading cells on MRS agar and counting the number of viable cells (Log CFU/ml) after incubation at 37°C for 48 h (Maragkoudakis et al., 2006). Tolerance to bile salts was evaluated by suspending cells in PBS solution containing 0.3% (w/v) bile salt (Sigma, St. Louis, MO, United States) and incubating at 37°C (Gilliland et al., 1984). Bile tolerance was measured using the same method used for acid tolerance.

#### Adhesion to Epithelial Cells

The adhesion assay was performed as described by Messaoudi et al. (2012). Caco-2 and HT-29 human colonic epithelial cell lines were obtained from the Korean Cell Line Bank (KCLB; Seoul, South Korea) and grown in Dulbecco’s modified Eagle’s medium (DMEM; Hyclone, Logan, UT, United States) supplemented with 10% fetal bovine serum (FBS; Hyclone), and 1% each of 10,000 U/mL penicillin and 10 mg/mL streptomycin (Hyclone) in 0.85% NaCl. The cells were cultured at 37°C in an atmosphere of 5% CO_2_ and 95% air, and the medium was changed regularly at 2 days intervals. All cells used in this study were between passages 37 and 40. Caco-2 and HT-29 cells were seeded in 24-well tissue culture plates (2 cm^2^ per well) at 4.7 × 10^5^ cells per well. Once the culture reached 80% confluency, the medium was changed to one without antibiotics. After cultivation of lactic acid bacteria in MRS medium, bacterial cells (10^8^ CFU/mL) were collected by centrifugation, washed twice with PBS (pH 7.4), and resuspended in DMEM without serum and antibiotics. The bacterial cells were then applied to a Caco-2 and HT-29 cell monolayer and incubated at 37°C in 5% CO_2_ for 2 h. After incubation, non-adherent bacteria were removed by washing twice with PBS. Cells with adhered bacteria were treated with a detachment solution containing 0.1% Triton X-100 and 0.1% trypsin-EDTA (Sigma) for 15 min. To calculate the number of adherent bacteria, the suspensions of the detached cells were plated onto MRS agar and incubated at 37°C for 48 h. The adhesion ability was estimated using the formula (the adhered bacteria/100 cells), where the Caco-2 and HT-29 cells were counted with hemocytometer (Thoma, Hirschmann, Germany).

#### Safety Assessment

To verify the safety of the strain, hemolysis analysis was conducted as described by Ryu and Chang (2013). Bacterial cells were inoculated into a BHI agar plate supplemented with 7% horse blood (MB CELL, Seoul, South Korea) and incubated under anaerobic conditions at 37°C for 24 h. Hemolysis was observed on the medium using *Listeria monocytogenes* as a positive control. In addition, the presence of biogenic amine genes in the genomic DNA of the selected strains was analyzed by multiplex PCR. The *hdc* (histidine decarboxylase) and *tyrdc* (tyrosine decarboxylase) genes were amplified using the following primer pairs: *hdc*: HDC3 (5-GATGGTATTGTTTCKTATGA-3) and HDC4 (5-CAAACACCAGCATCTTC-3); *tyrdc*: TD2 (5-ACATAGTCAACCATRTTGAA-3) and TD5 (5-CAAATGGA AGAAGAAGTAGG-3); 16S rRNA gene: 27F (5′-AGAGTTTGA TCMTGGCTCAG-3′) and 1492R (5′-GGTTACCTTGTT ACGACTT-3′) (O’Sullivan et al., 2015). In the multiplex PCR, each biogenic amine gene was amplified simultaneously with the 16S rRNA gene as a positive control for the PCR reaction in the tube by adding each corresponding primer set. Genomic DNA from bacterial strains was used as a template in the PCR reaction. The amplification program was as follows: 95°C for 5 min, followed by 32 cycles of 95°C for 45 s, 58°C for 45 s, and 72°C for 75 s, with a final extension at 72°C for 5 min. The genomic DNA of *L. reuteri* ATCC 23272 and *Enterococcus faecalis* KCCM 11729 were used as positive controls for the *hdc* and *tyrdc* genes, respectively.

#### Antioxidative Activity Assay

Antioxidative activity of the three fractions of bacterial cells was measured using the DPPH inhibition assay (Das and Goyal, 2015). For preparation of intact cells, cell-free extracts (CFE), and cell-free supernatant (CFS), bacteria were pre-cultured for 12 h, the main culture was performed for 12 h, and the optical density at 600 nm was adjusted to 1.0. The bacterial cells were washed twice and resuspended in 0.85% saline solution to obtain intact cells. For CFE, sonication was performed using a sonicator (VP-050N; Taitec Corp., Saitama, Japan) for 10 min (5 s on/5 s off pulse; at 35% amplitude), and the cell debris was removed by centrifugation (10,000 × *g* at 4°C for 5 min). CFS was prepared by centrifugation (10,000 × *g* at 4°C for 10 min) of the bacterial culture. The supernatant was neutralized (pH 7.0) with 1 M NaOH and passed through syringe filters to remove the remaining cells. The ethanolic DPPH solution (100 μL, 0.4 mM) was mixed with 100 μL of bacterial sample or water (control) and incubated at 37°C in the dark for 30 min. The absorbance of the mixture was measured at 517 nm using a microplate reader and compared with the reference compound, ascorbic acid (AA).

#### Nitric Oxide Assay

For the nitric oxide (NO) production assay, RAW 264.7 cells, a murine macrophage line, were obtained from the KCLB and maintained in DMEM supplemented with 10% FBS and 1% penicillin-streptomycin at 37°C in 5% CO_2_. To analyze the anti-inflammatory activity of bacterial strains, the effect of two bacterial fractions on the production of NO in LPS-induced RAW 264.7 cells was determined using Griess reagent as described by Yu et al. (2019). To prepare heat-killed bacteria, the absorbance of the strains at 600 nm was adjusted to 1.0, and the bacteria were heat-killed at 90°C for 30 min. After centrifugation at 10,000 × *g* for 5 min, the cell pellets were rinsed twice with PBS and suspended in DMEM. To prepare cell-free lysates, sonication was performed using a sonicator (VP-050N; Taitec Corp., Japan) for 10 min, and the cell debris was removed by centrifugation. The supernatant was filtered through a 0.22 μm microfilter membrane (polypropylene, Whatman, Kent, United Kingdom). RAW 264.7 cells (5 × 10^5^ cells per mL in 96-well plates) were pre-treated with Escherichia coli lipopolysaccharide (LPS; 1 μg/mL, Sigma) with or without heat-killed cells or cell-free lysate for 24 h. Then, the supernatant from each well was mixed with an equal volume of Griess reagent and placed in the dark for 10 min at room temperature. The absorbance of each well was measured at 540 nm using a microplate spectrophotometer (BioTek, Winooski, VT, United States). Nitrite levels in the growth medium were calculated using a standard curve constructed using NaNO_2_ in DMEM. Methyl arginine was used as a positive control to inhibit NO production.

#### Transepithelial Electrical Resistance Measurements

The epithelial barrier model and transepithelial electrical resistance (TEER) measurements were conducted using the method described by Zaylaa et al. (2018). Caco-2 cells were seeded on 12 well Transwell^®^ inserts (polyester membrane with 0.4 μm pore size, 12 mm diameter; Costar, Corning Life Science, Kennebunk, ME, United States) at a density of 5 × 10^4^ cells per cm^2^. The medium was changed every 2 days until confluency, when the optimal TEER value (≥200 Ω⋅cm^2^) was reached. TEER values were measured every 2 days using Millicell-ERS (Millipore, Billerica, MA, United States). Cells were then incubated in medium without antibiotics before experiments and treated in the insert with bacteria (10^8^ CFU/mL) 30 min prior to hydrogen peroxide (H_2_O_2_) administration (100 μM, to both the upper and lower sides). The TEER was measured every 30 min after sensitization. Results were expressed as% TEER compared with the initial TEER value at T_0_ (before the addition of H_2_O_2_) for each insert using the formula: TEER (Ω⋅cm^2^)/initial TEER (Ω⋅cm^2^) × 100 (%).

#### Intestinal Paracellular Permeability Using Fluorescein Isothiocyanate-Dextran

Intestinal permeability was tested using the fluorescein isothiocyanate (FITC)-dextran method described by Miyauchi et al. (2012) with slight modifications. The flux of FITC-dextran (molecular weight 4,000; FD4; Sigma, St. Louis, MO, United States) across the monolayer could be used as an indicator of cell permeability. After TEER measurement of H_2_O_2_-induced Caco-2 cells, FITC-dextran (100 μg/mL) was added to the upper chamber and placed in the dark for 4 h at room temperature. Aliquots (100 μL) of the sample were obtained from the lower chamber of each well and transferred to a black 96-well opaque plate. The fluorescence intensity was determined using a fluorescence spectrometer (LS55, Perkin Elmer Instruments, Waltham, MA, United States) at 485 nm and 535 nm, excitation and emission wavelengths, respectively. Cells not treated with bacteria were used as controls, and results were expressed as% of control.

### Starter Traits Analysis During Rice Fermentation

#### Rice Fermentation Condition

Rice solutions (5% and 10% w/v) were mixed with distilled water and sterilized at 121°C for 15 min. All strains were inoculated (10^7^ CFU/mL) in rice solution and incubated at 30°C for 24 h under anaerobic condition. After fermentation, samples were collected and stored at −20°C. The growth rate was measured by viable cell counts on MRS agar plate. The plates were incubated at 37°C for 48 h, and the viability was expressed as Log CFU/mL. The pH of each sample was monitored using a pH meter (Orion Versa, Thermo, United States).

#### Viscosity Analysis

The viscosity was measured at 17°C using an RVDV-II+Pro viscometer (Brookfield Engineering, Middleboro, MA, United States). Approximately 250 mL of each sample was placed in a glass beaker, and spindle no. 2–4 was used. Viscosity data were recorded at 30 s intervals and expressed in centipoise (cP). For comparison, commercial products such as fruit juice (orange, tomato), drinking yogurt (product A, product B), and semi-solid yogurt (plain, Greek) were analyzed together. Additionally, the reported viscosity data of sauce (a ketchup and a French mustard) were added from the ProSys Filling System web^1^.

#### Chemical Analysis

To conduct high-performance liquid chromatography (HPLC) analysis, a 5 mL sample was centrifuged at 10,000 × *g* for 10 min and the supernatant was filtered using 0.2 μm filters (Whatman, United Kingdom). Organic acids (lactic acid and acetic acid) were measured using HPLC 1260 Infinity (Agilent Technologies, United States) with Aminex HPX-87H column (300 × 7.8 mm; BioRad, CA, United States) under the operation setting: 0.008N H_2_SO_4_ in water as mobile phase, 20 μL of sample injection volume and 0.6 mL/min of flow rate. Sugars (glucose, maltose, maltotriose, maltotetrose, maltopentaose, and maltohexaose) were measured using HPLC Acme 9000 (Younglin, South Korea) with VN-50 4D column (150 × 4.6 mm; Shodex, Japan) under the operation setting: 67% acetonitrile (v/v) as mobile phase, 10 μL of sample injection volume and 0.3 mL/min of flow rate. Organic acids and sugars were detected using UV (215 nm) and RI detectors, respectively. Lactic acid, acetic acid (Wako, Japan), glucose, maltose (Junsei, Japan), maltotriose, maltotetrose, maltopentaose, and maltohexaose (TCI, Japan) were used as standards.

#### Enzyme Activity Assay

To prepare crude enzymes, bacteria were cultured at 37°C in 5% rice solution for 3 days and in MRS for 18 h, and the supernatants of bacterial cultures were harvested. For phytase activity, a modified Chalmers broth containing 1% sodium phytate (MCP) was used because the MRS medium contained high phosphate content. The cell-free supernatant fraction was collected by centrifugation at 10,000 × *g* for 10 min, and the methods were slightly modified from Giri et al. (2018). For α-amylase activity, 0.1 mL of supernatant was incubated with 0.1 mL of 1% starch (w/v) at 37°C for 30 min. The reaction was terminated using 0.6 mL of 3,5-dinitrosalicylic acid (DNS) reagent at 10 min intervals from the start of incubation, followed by boiling for 5 min. Absorbance at 540 nm was measured using a spectrophotometer (BioTek, United States). A standard curve was constructed using 2–10 mM glucose. One unit of amylase activity was defined as the amount of enzyme releasing 1 μmol of reducing sugars per minute under the assay conditions. For glucoamylase activity, 0.1 mL supernatant was incubated with 0.1 mL of 1% starch (w/v) at 37°C for 30 min. Then, 1 mL of a mixture of glucose oxidase/peroxidase reagent and o-dianisidine reagent in a glucose assay kit (GAGO20; Sigma-Aldrich, United States) was added at 10 min intervals from the start of incubation, followed by incubation at 37°C for 10 min. The reaction was terminated by adding 12 N H_2_SO_4_, and the absorbance was measured at 540 nm. A standard curve was constructed using 0.5∼2.5 mM glucose. One unit of glucoamylase activity was defined as the amount of enzyme that released 1 μmol glucose per minute under the assay conditions. For phytase activity, 0.1 mL) was mixed with 0.4 mL of substrate (3 mM sodium phytate in 0.2 M sodium acetate buffer, pH 4.0) and incubated at 37°C for 30 min. The reaction was stopped using 5% trichloroacetic acid (0.5 mL) at 10 min intervals from the start of incubation. Then, 0.5 mL of color reagent (1.5% (w/v) ammonium molybdate in 5.5% (v/v) sulfuric acid solution: 2.7% (w/v) ferrous sulfate solution = 1:4) was added. The absorbance at 700 nm was measured, and a standard curve was constructed using 2–10 mM KH_2_PO_4_. One unit of phytase activity was defined as the amount of enzyme releasing 1 nmol phosphate per minute under the assay conditions.

#### Metabolites Analysis by ^1^H-Nuclear Magnetic Resonance

Nuclear magnetic resonance (NMR) spectroscopy was used to analyze the metabolites produced in fermented rice solution. For the preparation of NMR samples, fermented rice was centrifuged, and the supernatant was collected. The pH of the supernatant was adjusted to 7.0 ± 0.2, by the addition of 5 N NaOH. The neutralized supernatant (350 μL) was mixed with 350 μL of 1 mM sodium trimethylsilyl propanesulfonate (DSS) as an internal standard in 10% D2O. The mixture (700 μL) was transferred to NMR tubes and subjected to ^1^H-NMR analysis. ^1^H-NMR spectra were recorded on an Avance 500-MHz spectrometer (Bruker BioSpin, Karlsruhe, Germany). The metabolite peaks in the NMR spectra were identified and their concentrations were calculated using the Chenomx NMR suite 8.4 library software (Chenomx Inc., Edmonton, AB, Canada).

### Microbial Characterization

Phylogenetic tree analysis of MG7011 was conducted by a maximum likelihood approach using MEGA: X (Molecular evolutionary genetics analysis) with other lactic acid bacteria (Kumar et al., 2018). Carbohydrate utilization ability of selected strains was analyzed using the API CHL kit (BioMériux Co., Marcy-l’Étoile, France) according to the manufacturer’s instructions. Bacterial cells were harvested after cultivation and resuspended in API 50CH medium. A 120 μL aliquot was inoculated into a tube of strips, and then mineral oil was dropped in the cupule to cover the tube, followed by incubation at 37°C for 48 h. Fermentation pattern was recorded as positive if the blue indicator in the medium changed to yellow; exceptionally, the color of tube number 25 changed to black. The enzyme activity profile of the selected strains was determined using API ZYM (BioMériux) according to the manufacturer’s instructions. Bacterial cells were harvested and resuspended in API resuspension medium. Then, the aliquot was inoculated into the cupule of strips and incubated at 37°C for 4 h. After incubation, reagents were added, and enzymatic activity was recorded as a numerical value ranging from 0 to 5, using the manufacturer’s color chart.

### Statistical Analysis

Each experiment was conducted in triplicate, and the data were presented as the mean value ± standard deviation (SD). Statistical analysis was performed using IBM SPSS software version 22 (SPSS Inc., United States). Independent *t*-tests were used to analyze differences between two groups, and one-way analysis of variance (ANOVA) with Tukey’s method was used to analyze differences between multiple groups. Different letters and symbols on the error bars indicate significant differences. Statistical significance was set at *P* < 0.05.

## Results

### Probiotic Properties of Isolates

#### Evaluation of Acid and Bile Salt Tolerance

The acid and bile tolerance levels of all strains were measured and compared with those of *L. rhamnosus* GG (LGG) as positive controls. *L. fermentum* DSM 20052 (LFT) and KCCM 35469 (LFC) were used as the type and commercial strains, respectively. As shown in Table 1, the viability of LGG decreased significantly after 90 min and 180 min of incubation at pH 2.5. In the case of LFT and LFC, their acid and bile tolerance levels also decreased. In contrast, most *L. fermentum* isolates showed higher viability than LGG, LFT, and LFC. Meanwhile, under bile salt conditions, only 10 strains (MG7011, MG4244, MG4261, MG5154, MG4500, MG4510, MG4531, MG4533, MG4535, and MG4536) showed higher levels of tolerance than the positive controls. Therefore, these 10 strains were selected for the next experiment.

**TABLE 1:** Viability (Log CFU/mL) of *Limosilactobacillus fermentum* strains in low pH conditions or 0.3% of bile salt.

Strains	Control	pH 3.0		pH 2.5		Bile salt 0.3%	
		90 min	180 min	90 min	180 min	90 min	180 min
LGG	8.53 ± 0.04	8.30 ± 0.10	8.27 ± 0.09	4.69 ± 0.10	4.31 ± 0.03	7.9 ± 0.05	6.22 ± 0.06
LFT	8.51 ± 0.04	7.91 ± 0.08	7.85 ± 0.12	6.36 ± 0.01	5.99 ± 0.36	4.8 ± 0.14	4.41 ± 0.06
LFC	8.68 ± 0.00	8.50 ± 0.03	8.35 ± 0.04	5.00 ± 0.04	3.67 ± 0.02	5.12 ± 0.18	5.07 ± 0.15
MG901	8.51 ± 0.07	8.31 ± 0.11	7.84 ± 0.10	7.24 ± 0.08	5.26 ± 0.03	7.13 ± 0.07	4.99 ± 0.09
MG7011	8.64 ± 0.02	8.34 ± 0.05	8.19 ± 0.04	7.28 ± 0.20	6.56 ± 0.05	7.87 ± 0.05	7.74 ± 0.07
MG7014	8.72 ± 0.07	8.66 ± 0.09	8.15 ± 0.12	7.70 ± 0.06	7.55 ± 0.06	6.19 ± 0.03	5.41 ± 0.07
MG4244	8.77 ± 0.02	8.73 ± 0.05	8.62 ± 0.02	8.55 ± 0.05	7.19 ± 0.08	8.47 ± 0.15	6.68 ± 0.04
MG4254	8.54 ± 0.02	8.45 ± 0.09	8.10 ± 0.05	8.26 ± 0.09	4.86 ± 0.03	7.42 ± 0.12	5.14 ± 0.18
MG4258	8.59 ± 0.06	8.39 ± 0.02	8.02 ± 0.05	8.26 ± 0.09	5.97 ± 0.16	5.14 ± 0.19	5.03 ± 0.11
MG4261	8.75 ± 0.10	8.57 ± 0.08	8.52 ± 0.04	8.30 ± 0.07	6.78 ± 0.04	8.58 ± 0.06	8.03 ± 0.06
MG4532	8.55 ± 0.05	8.43 ± 0.06	8.44 ± 0.04	8.25 ± 0.05	7.31 ± 0.08	7.44 ± 0.10	5.32 ± 0.17
MG4534	8.77 ± 0.08	8.54 ± 0.03	8.31 ± 0.01	8.31 ± 0.11	7.74 ± 0.03	6.06 ± 0.10	4.90 ± 0.22
MG4231	8.78 ± 0.05	8.64 ± 0.04	8.23 ± 0.07	7.35 ± 0.04	4.86 ± 0.03	8.43 ± 0.05	5.66 ± 0.07
MG5154	8.63 ± 0.04	8.24 ± 0.07	8.16 ± 0.03	8.27 ± 0.10	4.31 ± 0.05	8.33 ± 0.08	7.37 ± 0.06
MG4500	8.59 ± 0.05	8.29 ± 0.19	8.05 ± 0.04	8.21 ± 0.14	6.59 ± 0.04	8.25 ± 0.05	7.83 ± 0.04
MG4510	8.60 ± 0.14	7.82 ± 0.08	7.82 ± 0.07	6.61 ± 0.16	4.21 ± 0.07	7.31 ± 0.10	7.26 ± 0.03
MG4529	8.65 ± 0.07	8.31 ± 0.03	5.07 ± 0.08	4.80 ± 0.08	4.64 ± 0.02	5.07 ± 0.18	4.16 ± 0.26
MG4530	8.58 ± 0.06	8.28 ± 0.11	8.15 ± 0.04	8.02 ± 0.12	6.49 ± 0.04	7.23 ± 0.07	5.33 ± 0.06
MG4531	8.50 ± 0.06	8.40 ± 0.16	8.34 ± 0.11	7.74 ± 0.05	6.44 ± 0.04	7.4 ± 0.10	7.34 ± 0.07
MG4533	8.64 ± 0.05	8.57 ± 0.06	8.36 ± 0.03	8.36 ± 0.07	5.44 ± 0.06	8.32 ± 0.02	7.67 ± 0.05
MG4535	8.60 ± 0.10	8.53 ± 0.04	7.44 ± 0.02	7.67 ± 0.04	4.19 ± 0.09	8.09 ± 0.04	6.65 ± 0.08
MG4536	8.59 ± 0.05	8.47 ± 0.10	7.17 ± 0.02	7.60 ± 0.09	4.17 ± 0.07	8.48 ± 0.12	7.74 ± 0.10
MG4538	8.65 ± 0.09	7.84 ± 0.12	6.65 ± 0.45	7.66 ± 0.09	5.90 ± 0.09	4.92 ± 0.20	4.63 ± 0.14
MG4539	8.74 ± 0.07	8.24 ± 0.42	7.01 ± 0.04	7.40 ± 0.08	4.47 ± 0.06	7.16 ± 0.16	5.73 ± 0.05
MG4540	8.68 ± 0.20	8.46 ± 0.15	7.76 ± 0.09	7.44 ± 0.06	4.18 ± 0.05	5.25 ± 0.36	4.66 ± 0.03
MG4542	8.70 ± 0.15	7.95 ± 0.13	7.70 ± 0.04	7.92 ± 0.05	4.03 ± 0.06	3.70 ± 0.00	4.11 ± 0.13
MG4545	8.71 ± 0.20	8.14 ± 0.04	6.27 ± 0.04	5.05 ± 0.08	4.58 ± 0.05	5.76 ± 0.09	5.29 ± 0.17
MG5341	8.75 ± 0.25	8.42 ± 0.02	6.01 ± 0.27	5.97 ± 0.05	4.44 ± 0.03	4.81 ± 0.02	4.53 ± 0.10

*Data are the mean ± SD (n = 3).*

*LGG, Lacticaseibacillus rhamnosus GG; LFT, Limosilactobacillus fermentum DSM 20052^T^; LFC, Limosilactobacillus fermentum KCCM 35469.*

#### Adhesion to Epithelial Cells

**FIGURE 1:**
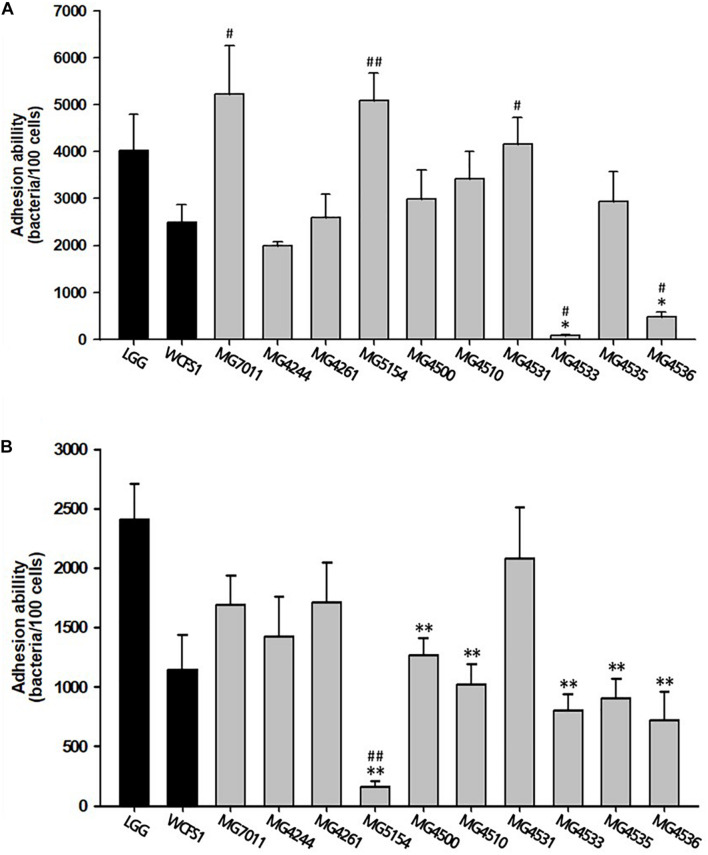
Intestinal adhesion ability of *Limosilactobacillus fermentum* strains. **(A)** Caco-2 cells, **(B)** HT-29 cells as colonic epithelial cells. Data are the mean ± SD (*n* = 3). ^∗^*p* < 0.05, ^∗∗^*p* < 0.01 compared with control strain WCFS1 and ^#^*p* < 0.05, ^##^*p* < 0.01 compared with the control strain LGG.

The adhesion ability of the selected strains to intestinal epithelial cells was measured by incubating bacterial cells with epithelial cell lines, Caco-2 and HT-29 cells. As shown in Figure 1A, three strains (MG7011, MG5154, and MG4531) showed higher adhesion to Caco-2 cells than WCFS1 (4000 CFU/100 cells) and five strains (MG4244, MG4261, MG4500, MG4510, and MG4535) showed high adhesion ability comparable to LGG (2500 CFU/100 cells). As shown in Figure 1B, only four strains (MG7011, MG4244, MG4261, and MG4531) showed high adhesion abilities comparable to those of WCFS1 (2400 CFU/100 cells) and LGG (1100 CFU/100 cells). Consequently, the five strains (MG7011, MG4244, MG4261, MG4500, and MG4531) were selected for good adhesion to intestinal epithelial cells, Caco-2 and HT-29.

#### Hemolytic Activity and Biogenic Amine Genes

**FIGURE 2:**
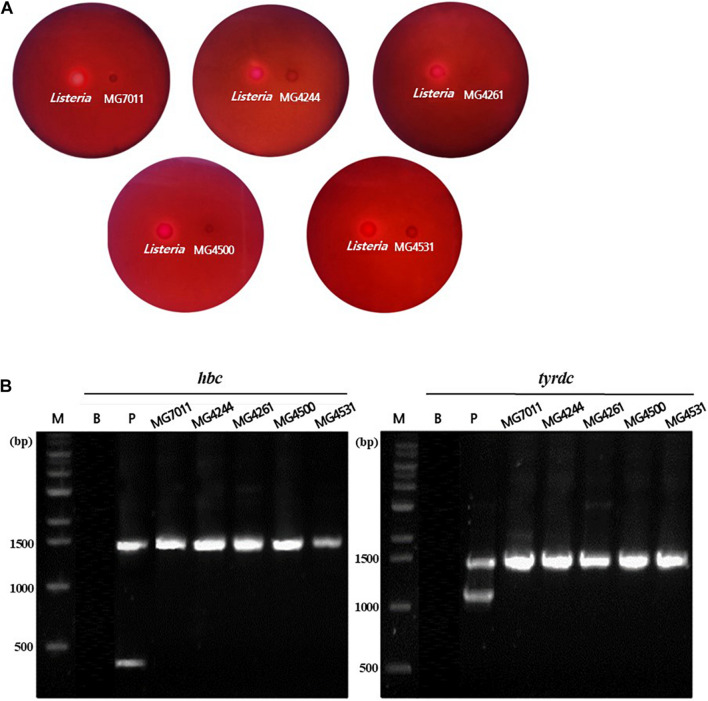
Safety assessment of selected *Limosilactobacillus fermentum* strains. **(A)** Hemolytic activity analysis of *L. fermentum* strains. Hemolytic activity was measured in BHI broth containing 7% horse blood. Left, positive control, *Listeria monocytogenes*, showing clear zone around the cell drop; right, the MG7011, MG4244, MG4261, MG4500, and MG4531, respectively. **(B)** Detection of genes related to biogenic amine production. lane M, 1 kb DNA marker; lane B, negative control, which has no template DNA; lane P, positive controls having *hdc* (histidine decarboxylase, 440 bp), and *tyrdc* (tyrosine decarboxylase, 1100 bp) genes from *Limosilactobacillus reuteri* ATCC 23272 and *Enterococcus faecalis* KCCM 11729, respectively. The DNA of the 16S rRNA gene (1530 bp) was also amplified.

To evaluate the safety of *L. fermentum* strains, the hemolytic activity and biogenic amine genes were analyzed. As shown in Figure 2A, the five strains selected above, MG7011, MG4244, MG4261, MG4500, and MG4531, did not show any clear zones around the cell drop, whereas *Listeria monocytogenes* as positive control showed a clear zone that could be interpreted as hemolytic activity. In addition, as shown in Figure 2B, *hdc* and *tydc* genes were not detected in the five selected strains which produce histamine and tyramine, respectively, but clear bands appeared in positive controls. These results reveal that selected strains are considered as safe with non-hemolytic activity and no *hdc* and *tyrdc* genes.

#### Antioxidative Activity

**FIGURE 3:**
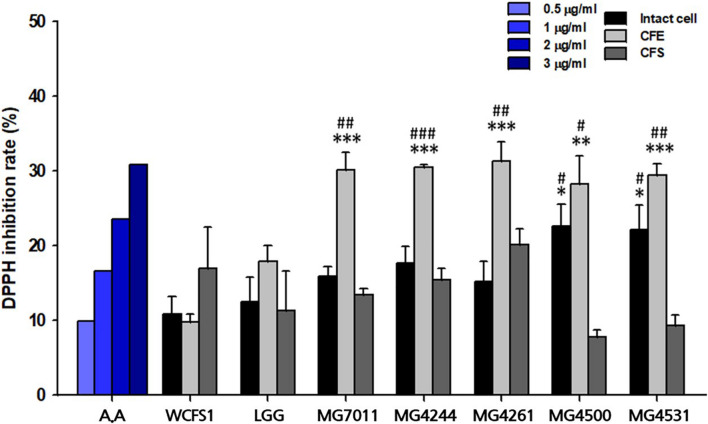
Antioxidative activity of *Limosilactobacillus fermentum* strains. A represents ascorbic acid. *Limosilactobacillus plantarum* WCFS1 (WCFS1) and *Limosilactobacillus rhamnosus* GG (LGG) were used as positive controls. Data represent the mean ± SD (*n* = 3). ^∗^*p* < 0.05, ^∗∗^*p* < 0.01, ^∗∗∗^*p* < 0.001 compared with control strain WCFS1 and ^#^*p* < 0.05, ^##^*p* < 0.01, ^###^*p* < 0.001 compared with control strain LGG.

To evaluate antioxidative activity, DPPH scavenging capacities of isolates were measured by using intact cells, cell-free extracts (CFE), and cell-free supernatants (CFS). In case of intact cells, all *L. fermentum* strains exhibited higher DPPH scavenging activity (>15%) than positive control, LGG (12.4%) as shown in Figure 3. Among them, MG4500 and MG4531 showed the highest DPPH scavenging activity (22.6% and 22.1%, respectively). In the case of CFE, all *L. fermentum* strains showed >28% activity, which was significantly higher than that of LGG (17.9%). In addition, all strains showed similar levels of DPPH scavenging activity as LGG when CFS were used. These results show that the five selected strains of *L. fermentum* have excellent antioxidative activity compared to LGG and WCFS1.

#### Measurement of Nitric Oxide Production

**FIGURE 4:**
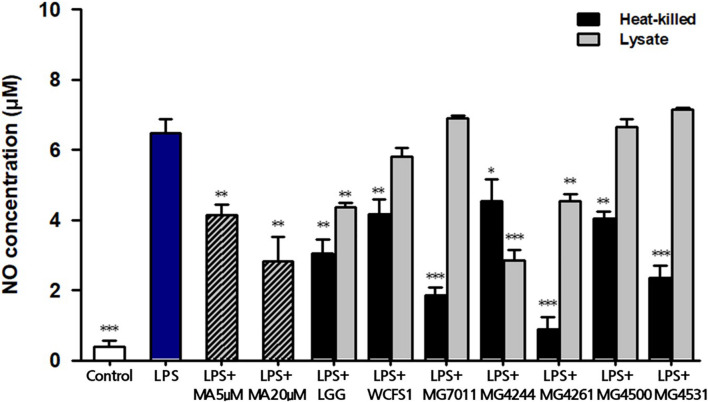
Inhibitory activity of heat-killed and lysate forms of *Limosilactobacillus fermentum* cells on nitric oxide (NO) production in LPS-induced RAW264.7. MA, methyl arginine was used as positive control. Data represent the mean ± SD (*n* = 3). ^∗^*p* < 0.05, ^∗∗^*p* < 0.01, ^∗∗∗^*p* < 0.001 compared with LPS.

To evaluate the anti-inflammatory activities of the selected strains, the inhibitory activities of heat-killed and lysate cells were tested on NO production in LPS-induced RAW 264.7 cells. As shown in Figure 4, treatment with LPS markedly increased the NO production (6.47 ± 0.40 μM), compared with the control that was not treated with LPS (0.39 ± 0.19 μM). In addition, treatment with 5 μM and 20 μM methyl arginine, which is a nitric oxide synthase inhibitor, inhibited the production of NO in a dose-dependent manner (4.15 ± 0.28 μM and 2.82 ± 0.71 μM, respectively). In cases of heat-killed strains, all tested samples including LGG and WCFS1 exhibited significant inhibitory activities on NO production compared with the LPS-treated group (*p* < 0.05). Particularly, three heat-killed strains (MG7011, MG4261, MG4531; <2.4 μM of NO) showed significantly stronger inhibitory activity than LGG (3.0 μM). In lysates, LGG, MG4244, and MG4261 (3.8 μM, 2.8 μM, and 4.5 μM, respectively) showed significant inhibitory effects compared with the LPS-treated group (*p* < 0.01 for LGG and MG4261, *p* < 0.001 for MG4244).

#### Protective Effects on Hydrogen Peroxide-Induced Intestinal Permeability

**FIGURE 5:**
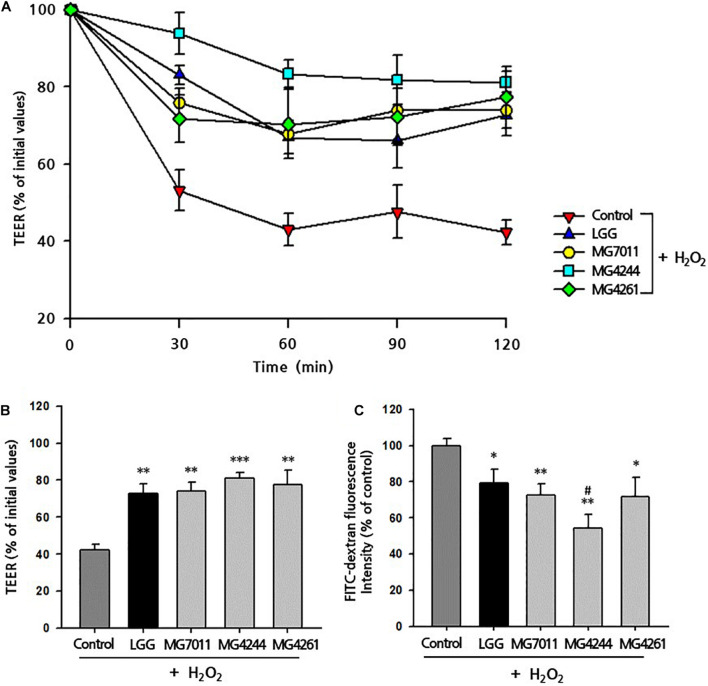
Preventive effect of *Limosilactobacillus fermentum* strains on H_2_O_2_-induced permeabilization of Caco-2 cell monolayers. Caco-2 cell monolayers were pretreated with bacterial cells for 30 min, and the monolayers were exposed to H_2_O_2_ (100 μM). TEER was measured **(A)** every 30 min **(B)** until 2 h after H_2_O_2_ treatment. FITC-dextran fluorescence intensity **(C)** was measured 4 h after TEER measurement and expressed in% compared with control value. *Limosilactobacillus rhamnosus* GG (LGG) was used as a positive control. All data are expressed as mean ± SD (*n* = 3). ^∗^*p* < 0.05, ^∗∗^*p* < 0.01, ^∗∗∗^*p* < 0.001 compared with control and ^#^*p* < 0.05 compared with LGG.

To test the protective activities of selected strains for epithelial monolayers, bacterial cells and H_2_O_2_ were incubated with Caco-2 cells after confluency, and the TEER value and FITC-dextran flux were measured. As shown in Figure 5A, the addition of H_2_O_2_ to Caco-2 cell monolayers caused a decrease in TEER (42.34%) along with incubation time, but LGG and the three selected strains significantly protected against H_2_O_2_-induced epithelial damage. In Figure 5B, the protective effects of MG7011, MG4244, and MG4261 were compared, which resulted in an increase in TEER (74.06%, 81.15%, and 77.36%, respectively) compared to LGG (72.64%) after 120 min. Additionally, in Figure 5C, the paracellular permeability of Caco-2 cell monolayers was measured using FITC-dextran flux. Each well was incubated with FITC-dextran for 4 h, and the fluorescence transmitted through Caco-2 cells was measured as% based on the control (treated with only H_2_O_2_). All bacterial strains showed lower fluorescence (<79.3%) than the control group (*p* < 0.01), and MG4244 showed the lowest values (54.2%) compared to LGG (79.2%) (*p* < 0.05). The results show that MG7011, MG4244, and MG4261 strains have protective activities for epithelial cells against oxidative stress induced by H_2_O_2_ and lower the permeability of Caco-2 cell monolayers. In conclusion, the three strains, MG7011, MG4244, and MG4261, were selected as potential probiotic candidates.

### Starter Properties of Isolates

#### Rice Fermentation by Selected Strains

**FIGURE 6:**
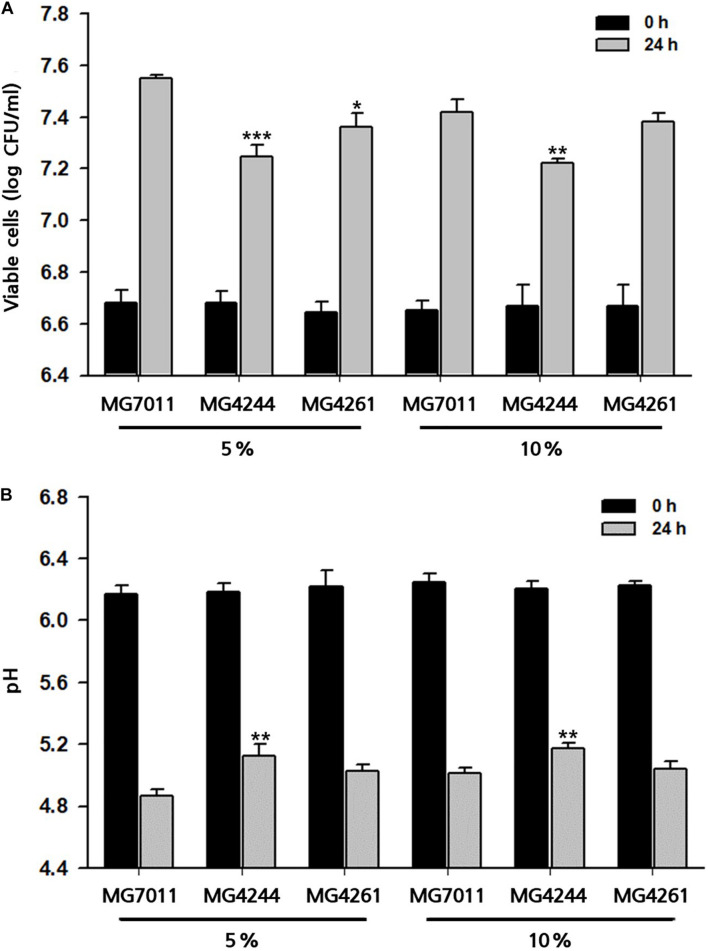
Growth profiles of selected *Limosilactobacillus fermentum* strains 5% and 10% rice at 30°C for 24 h. **(A)** viable cells counts and **(B)** pH value. Results are expressed as means ± SD (*n* = 3). *p < 0.05, **p < 0.01, ***p < 0.001 compared with MG7011.

**FIGURE 7:**
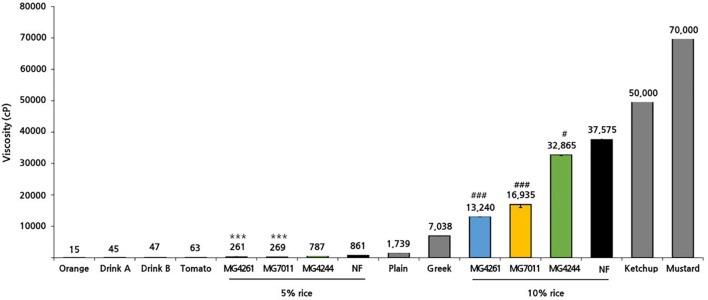
Viscosity of rice solution fermented by *Limosilactobacillus fermentum* strains. Viscosities of fermented rice yogurt and commercial products were measured at 17°C. Results are expressed as means ± SD (*n* = 3). There were significant differences compared with 5% non-fermented rice (NF; ***p < 0.001) and 10% non-fermented rice (NF; ^#^*p* < 0.05, ^###^*p* < 0.001). For comparison, commercial products such as fruit juice (orange, tomato), drinking yogurt (product A, product B), and semi-solid yogurt (plain, Greek) were analyzed together.

To prepare different types of fermented rice, such as drink or semi-solid type, 5% and 10% of rice solutions were fermented by the selected strains at 30°C for 24 h, and fermentative properties (viable cell, pH, and viscosity) were measured. As shown in Figure 6, three strains grew well in both 5% and 10% rice solutions from the initial cell count of 6.7 Log CFU/mL to more than 7.2 Log CFU/mL after 24 h – especially, MG7011 showed superior cell growth. The scales of increase in viable cells and decrease in pH for each strain were consistent. Meanwhile, as shown in Figure 7, 5% rice solution fermented with MG7011, MG4244, or MG4261 showed lower viscosity levels respectively (269, 787, and 261 cP, respectively) than non-fermented rice (NF, 861 cP). In contrary, 10% rice solution fermented with three strain showed higher viscosity than that of 5% rice. In the case of 5% fermented rice, viscosities were observed between the two groups of commercial products as follows: higher than several drinking beverages (orange juice, tomato juice, drink A, and drink B), but lower than plain or Greek yogurts. In contrast, viscosities of 10% fermented rice were higher than plain or Greek yogurts and lower than commercial ketchup or mustard products. In summary, these results show that the viscosities of fermented rice can be controlled by the initial concentration of rice in between 5% and 10%, and MG7011 has the highest growth rate and pH change in both 5% and 10% rice solutions, revealing its good adaptability in rice medium.

#### Enzyme Activities Expressed in Selected Strains

Enzyme activities related to rice utilization were analyzed against MG7011, MG4244, and MG4261: α-amylase and glucoamylase to hydrolyzed starch, and phytase to hydrolyzed phytic acid (Table 2). In the case of α-amylase, MG7011 showed activity in 5% rice solution (0.11 U) and MRS (0.08 U), while MG4261 showed lower levels in MRS (0.02 U). In the case of glucoamylase, three strains showed 0.01∼0.02 U in rice solution. In the case of phytase, MG7011 showed activity in MCP (17.65 U). This result revealed that the MG7011 strain can produce α-amylase, glucoamylase, and phytase.

**TABLE 2:** Enzyme activities of *Limosilactobacillus fermentum* strains cultured in different media.

Strains	α-Amylase		Glucoamylase		Phytase		
	5% rice	MRS	5% rice	MRS	5% rice	MRS	MCP
MG7011	0.11	0.08	0.02	−	−	−	17.65
MG4244	−	−	0.01	−	−	−	−
MG4261	−	0.02	0.01	−	−	−	−

*Supernatants of bacterial cultures were used for activity assay. Bacteria were cultured in 5% rice solution for 3 days, and in MRS and modified Chalmers broth containing 1% sodium phytate (MCP) for 18 h both at 37°C. Negative (−) symbol denotes no enzyme activity detected.*

#### Metabolites Analysis by ^1^H-Nuclear Magnetic Resonance

**FIGURE 8:**
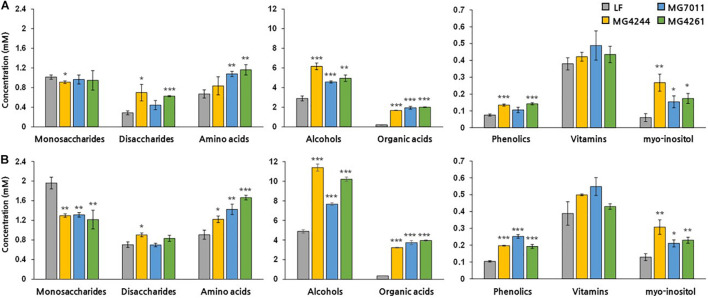
Metabolite concentrations (mM) in fermented rice by *Limosilactobacillus fermentum* strains. Significant differences in **(A)** 5% and **(B)** 10% rice were indicated as *p < 0.05, **p < 0.01, ***p < 0.001 compared with non-fermented rice (NF).

To analyze the changes in various metabolites during rice fermentation, NMR analysis was conducted. As shown in Figure 8, after fermentation of 5% and 10% rice solution by MG7011, MG4244 and MG4261 for 24 h, the concentrations of amino acids, alcohols, organic acids, phenolics, and myo-inositol increased. In the case of sugars, glucose, fructose, and maltose increased, and sucrose decreased in fermented rice by all strains. Sucrose in rice can be hydrolyzed into fructose and glucose, and they are converted into several products such as lactate, acetate, ethanol, and glucitol. Maltose is produced by the bacterial amylase from rice starch. All strains produced significant amounts (>2 folds) of γ-aminobutyric acid (GABA). As compared in Supplementary Figure 2, in amino acids, glycine and histidine decreased, and proline, threonine, and alanine increased due to bacterial metabolism. Most samples showed significant increases in certain phenolic compounds, including 4-hydroxybenzoate, chlorogenate, ferulate, and vanillate. In particularly, myo-inositol was produced by all strains, and MG7011 resulted in 4.4 folds higher synthesis in 5% rice. Indeed, MG7011 efficiently hydrolyzed phytic acid as shown in Table 2. Metabolite patterns in the 5% and 10% rice solutions were generally consistent. Based on the results obtained in rice fermentation as well as probiotic traits, we selected *L. fermentum* MG7011 as the best strain for probiotic starter for fermentation of rice beverage.

### Microbial Characteristics of MG7011

To identify the microbial characteristics, phylogenetic tree, carbohydrate utilization, and enzyme activity patterns were analyzed. First, its phylogenetic tree analysis with other lactic acid bacteria showed MG7011 belonging to *L. fermentum* group with 99.93% identity with the type strain, *L. fermentum* DSM 20052 (Supplementary Figure 1). Next, carbohydrate utilization patterns of MG7011 were compared with the type strain, *L. fermentum* DSM 20052. As a result (Supplementary Table 1), MG7011 and DSM 20052 commonly utilized D-ribose, D-galactose, D-glucose, D-fructose, D-maltose, D-lactose, and D-sucrose. However, notably, MG7011 could utilize L-arabinose, mannose, melibiose, raffinose, and gluconate that the type strain could not use, showing 91.3% identity with the type strain when analyzed on the API web^2^. In addition, as shown in Supplementary Table 2, MG7011 showed various enzyme activities, such as lipid hydrolyzing enzymes (esterase and esterase-lipase), peptidase (leucine valine arylamidase), phosphatase (acid phosphatase and naphthol-AS-BI-phosphohydrolase), and galactosidase (α- and β-). These results indicate that newly isolated MG7011 is a novel strain with unique carbohydrate utilization pattern and various enzyme activity. The final selected strain was deposited in the Korean Agricultural Culture Collection (KACC) with accession no. KACC 81147BP.

## Discussion

Cereals such as rice, barley, and oats have gained attention as popular items for consumption owing to the various drawbacks associated with dairy products. To meet this demand, fermented cereal beverages supplemented with probiotics have been produced (Maria et al., 2020), and previous studies have attempted to develop probiotic starters for rice (Ghosh et al., 2015; Giri et al., 2018). Rice generally contains approximately 70% starch, 8% protein, 3% fat, and micronutrients (Choe et al., 2002). It is known that rice can promote the growth of beneficial bacteria, *Lactobacillus* (Kedia et al., 2007) and *Bifidobacterium* (Rozada-Sánchez et al., 2008) and help to increase their viability under gastrointestinal conditions (Charalampopoulos et al., 2003; Michida et al., 2006). Thus, starch and fiber in rice can be used as a carbon source and an effective prebiotic for lactic acid bacteria. In this study, *L. fermentum* MG7011 was selected for its excellent probiotic and starter properties for rice fermentation.

First, *L. fermentum* MG7011 exhibited excellent probiotic properties. To colonize the intestine, LAB must be stable in the human gastrointestinal tract and adhere to epithelial cells. MG7011 strain was higher tolerant in pH 2.5 (≥6.6 Log CFU/mL) and 0.3% bile (>7.7 Log CFU/mL) than commercial strains, LGG (4.3 and 6.2 Log CFU/mL, respectively) and LFC (3.7 and 5.1 Log CFU/mL, respectively) (Table 1). MG7011 also adhered well to intestinal epithelial cells, Caco-2 and HT-29, comparable to WCFS1 (Figure 1) and higher Caco-2 adhesion (5,200 CFU/100 cells) than LGG (2,500 CFU/100 cells), *p* < 0.05). Moreover, MG7011 had several health-promoting effects; the bacterial fraction showed antioxidant activities; in particular, the cell-free extract exhibited high activity (>30% DPPH radical scavenging), which is equivalent to 3 μg/mL of ascorbic acid (Figure 3). The bacterial fraction also inhibited NO production in LPS-induced macrophage cells, comparable to the NO synthase inhibitor methyl arginine (Figure 4). Notably, MG7011 protected intestinal cells against oxidative stress and H_2_O_2_ (Figure 5).

Second, *L. fermentum* MG7011 exhibited excellent starter properties. Amylolytic enzymes are required for rice carbohydrate utilization, and some strains have been observed in *Lactobacillus* and *Bifidobacterium* (Espirito-Santo et al., 2014). In this study, *L. fermentum* MG7011 grew well in rice at the 30°C temperature (Figure 6) because it hydrolyzed starch and utilized sugars for its growth (Espirito-Santo et al., 2014). MG7011 showed α-amylase, glucoamylase (Table 2), and α-glucosidase activity (Supplementary Table 2), which degrade starch and oligosaccharides by endo and exo action. The strain increased maltose content in fermented rice (Supplementary Figure 2) and produced malto-oligosaccharides (data not shown). Patterns of each mono- and disaccharides change (Figure 8) was consistent with previous report of rice beverage ‘calugi’ after 12 h fermentation (Miguel et al., 2012). In addition, the MG7011 strain increased amino acid concentrations (Figure 8), which is related to peptidase activity (Supplementary Table 2); it showed high leucine arylamidase activity, with leucine increase in 10% rice, but not significantly in 5% (Supplementary Figure 2). Meanwhile, the increased phenolic compounds in fermented rice (Figure 8) could be attributed to esterase activity (Supplementary Table 2), which hydrolyses complex forms and releases phenolics (Adebo and Gabriela Medina-Meza, 2020). Higher concentrations of phenolics and flavonoids in fermented rice could lead to increased antioxidant activity and DPPH and ABTS scavenging activities (Giri et al., 2018). Phytic acid in rice forms complexes with minerals and inhibits its bioavailability (Mabunga et al., 2015), but bacterial phosphatases, including phytase, can degrade phytic acid and increase mineral concentration during fermentation (Espirito-Santo et al., 2014). In this study, *L. fermentum* MG7011 showed phosphatase activity and the highest phytase activity (Table 2). Indeed, MG7011-fermented rice showed increased levels of myo-inositol (Figure 8), which is consistent with previous studies. Meanwhile, *L. fermentum* MG7011 lowered the viscosity of the fermented rice (Figure 7). After cooling the gelatinized starch, amylose connects to double-helix aggregates to form a gel network (Majzoobi and Beparva, 2014). In rice fermentation, bacterial enzymes hydrolyzed starch to soluble oligosaccharides (Phattra and Maweang, 2015) and organic acids interrupt hydrogen bonds and the helical structure of starch, causing a weak molecular structure (Ačkar et al., 2015). Bacterial exopolysaccharides (EPS) are another important factor in viscosity. Since several EPS-producing *L. fermentum* strains have been reported (Ale et al., 2020), further studies are needed for EPS production of the strain and its effect on viscosity in fermented rice.

Fermented foods are delivery vehicles for probiotics and have desirable health-promoting effects (Tamang et al., 2020). A previous study reported that bacterial culture and fermented foods provide equal levels of functionality. In piglets, each group fed either Propionibacterium freudenreichii (PF-culture) alone or cheese fermented with the strain (PF-cheese) showed 10^7^ CFU/g of propionibacteria population and increased levels of total short-chain fatty acids in feces. In addition, PF-culture and PF-cheese were associated with low concentrations of IL-10 and TNF-α cytokines in piglet PBMCs, and increased IL-10 in LPS-induced piglet PBMCs, suggesting that these probiotic administrations modulate immunity (Rabah et al., 2018). This study confirmed that *L. fermentum* MG7011 has beneficial probiotic activities, grows well in rice, and produces health-promoting metabolites such as phenolics and vitamins. Therefore, the intake of fermented rice with *L. fermentum* MG7011 will enable the delivery of not only a beneficial probiotic but also a health-enhancing compound. Further studies are needed to determine the precise health-promoting activities of selected strains and their fermented rice products.

## Data Availability Statement

The original contributions presented in the study are included in the article/Supplementary Material, further inquiries can be directed to the corresponding author.

## Author Contributions

YJ performed experiments/data collection and drafted the manuscript. GK performed experiments/data collection. S-AK provided stylistic/grammatical revisions to manuscript. SC analyzed the data. C-HK provided revisions to scientific content. NH was a principal investigator (advisor, head of project, and manager). All authors contributed to the article and approved the submitted version.

## Conflict of Interest

C-HK is employed at the MEDIOGEN, Co., Ltd. The remaining authors declare that the research was conducted in the absence of any commercial or financial relationships that could be construed as a potential conflict of interest.

## Publisher’s Note

All claims expressed in this article are solely those of the authors and do not necessarily represent those of their affiliated organizations, or those of the publisher, the editors and the reviewers. Any product that may be evaluated in this article, or claim that may be made by its manufacturer, is not guaranteed or endorsed by the publisher.

## Footnotes

1
https://prosysfill.com/product-viscosity-chart

2
https://apiweb.biomerieux.com
