# Supplementary material for: Limosilactobacillus fermentum MG7011: An Amylase and Phytase Producing Starter for the Preparation of Rice-Based Probiotic Beverages
Source: Front Microbiol. 2021 Sep 29;12:745952. doi: 10.3389/fmicb.2021.745952 (PMC8511794; doi:10.3389/fmicb.2021.745952)
Supplement: Supplementary file 1 [file Data_Sheet_1.docx]

Supplementary Material

**Supplement Table 1. Carbohydrate utilization profile of selected *Limosilactobacillus fermentum* MG7011^a^**

| Carbohydrate | MG7011 | DSM 20052^b^ |
| --- | --- | --- |
| L-arabionose | **+** | ̶ |
| D-Mannose | **+** | ̶ |
| D-Melibiose | **+** | ̶ |
| Trehalose | **+** | ̶ |
| D-Raffinose | **+** | ̶ |
| Gluconate | **+** | ̶ |
| 5-ketogluconate | **+** | ̶ |

^a^MG7011 strain was positive for D-ribose, D-galactose, D-glucose, D-fructose, D-maltose, D-lactose, and D-sucrose. MG7011was negative for glycerol, erythritol, D-arabinose, D-xylose, L-xylose, D-adonitol, methyl-β-D-xylopyranoside, L-sorbose, L-rhamnose, dulcitol, inositol, D-mannitol, D-sorbitol, methyl-α-D-mannopyranoside, methyl-α-D-glucopyranoside, N-acetyl-glucosamine, amygdaline, arbutin, esculin, salicin, D-cellobiose, inulin, D-melezitose, starch, glycogen, xylitol, gentibiose, D-turanose, D-lyxose, D-tagatose, D-fucose, L-fucose, D-arabitol, L-arabitol, and 2-ketogluconate. ^b^The results of *L. fermentum* DSM 20052 were referenced from API test finder (https://bacdive.dsmz.de/api-test-finder).

**Supplement Table 2. Enzyme activity profile of selected *Limosilactobacillus fermentum* MG7011**

| Enzyme | Activity level^a^ |
| --- | --- |
| Esterase (C4) | 4 |
| Esterase-Lipase (C8) | 1 |
| Leucine arylamidase | 4 |
| Valine arylamidase | 2 |
| Cysteine arylamidase | 0 |
| Acid phosphatase | 2 |
| Naphtol-AS-BI-phosphohydrolase | 3 |
| α-Galactosidase | 5 |
| β-Galactosidase | 5 |
| α-Glucosidase | 5 |

Enzyme activity profile was determined using the API ZYM (BioMériux) according to the manufacturer's instruction. ^a^A numerical value ranging from 0 to 5 was assigned according to the color chart provided by the manufacturer; negative = 0, weak positive = 1 or 2, and positive = 3, 4, or 5; MG7011 was negative for alkaline phosphatase, lipase (C14), trypsin, α-chymotrypsin, β-glucuronidase, β-glucosidase, N-acetyl-β-glucosaminidase, α-mannosidase, and α-fucosidase.

**Supplement Figure 1. Phylogenetic tree of *Limosilactobacillus fermentum* MG7011 obtained in this study.**

Neighbour-joining phylogenetic tree, based on 16S rRNA gene sequences, showing the relationships between MG7011 and members of the genus *Limosilactobacillus* and *Lactoplantibacillus*. *Lactoplantibacillus plantarum* WCFS1was used as an outgroup. Bar, 0.005 nucleotide substitutions per site.


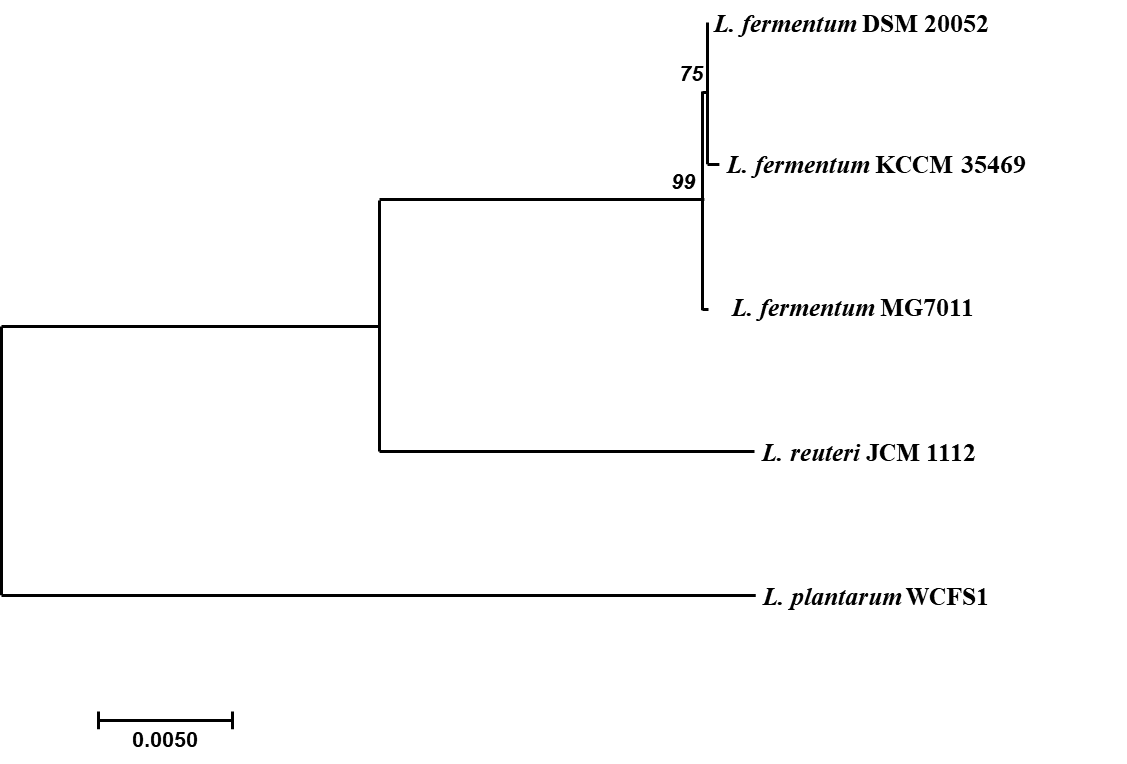


**Supplement Figure 1.**

**Supplement Figure 2. Metabolite changes (log2) in fermented rice yogurt by *Limosilactobacillus fermentum* strains** Compounds in (A) 5% and (B) 10% rice with a significant decrease or increase (*p* < 0.05) are shown in red and blue, respectively.


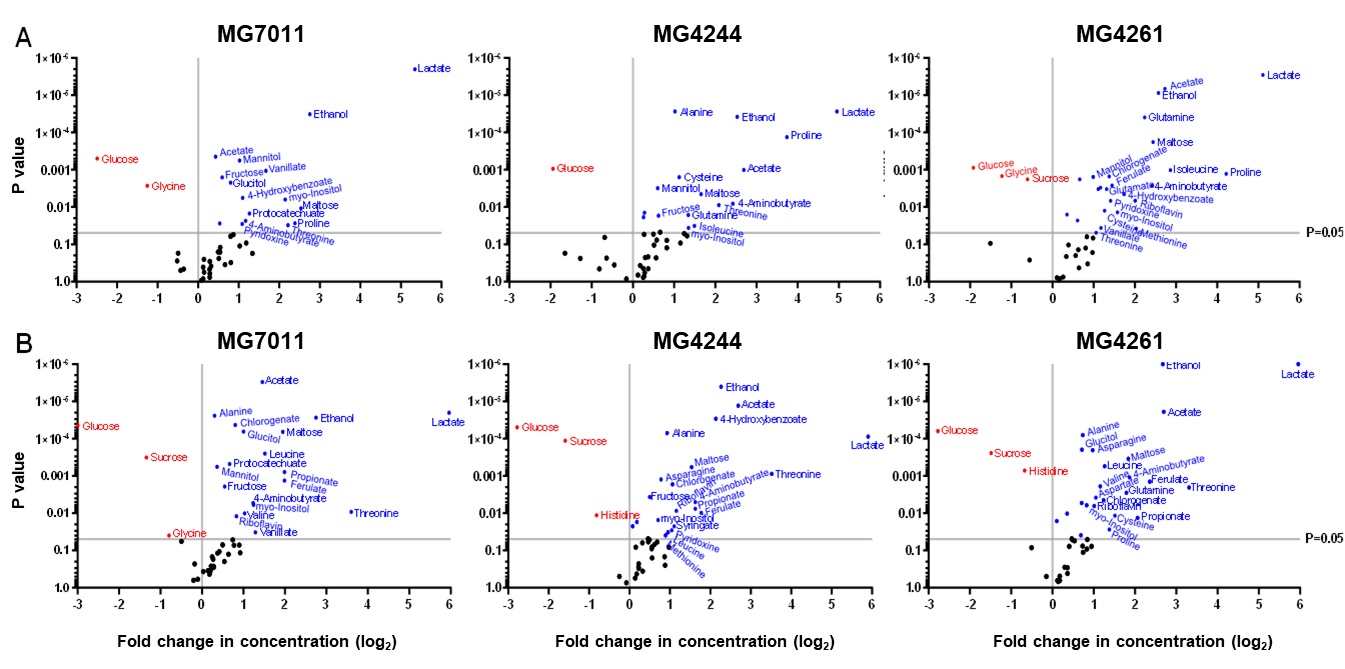


**Supplement Figure 2.**
